# Under‐Served Groups and Myalgic Encephalomyelitis Research Workshop; Multiple Barriers to Effective Healthcare, Research and Public Participation

**DOI:** 10.1111/hex.70214

**Published:** 2025-03-17

**Authors:** Monica Jane Bolton, Carolyn A. Chew‐Graham, Harm van Marwijk

**Affiliations:** ^1^ Brighton and Sussex Medical School Brighton East Sussex UK; ^2^ School of Medicine, Keele University, Staffs UK; ^3^ Brighton and Sussex Medical School, Primary and Community Health Services Lead ARC KSS UK

**Keywords:** chronic fatigue syndrome, myalgic encephalomyelitis, public participation, under‐served groups, workshop

## Abstract

Public involvement in research and other initiatives for myalgic encephalomyelitis (ME) (also known as chronic fatigue syndrome) has been crucial in raising awareness of the disease and exposing inadequate healthcare and research funding. An online workshop on ME research and under‐served groups took place in July 2024, organised by the first author, a person with ME. The workshop illustrated very low prevalence and thus barriers to healthcare as well as limited research in people from under‐served groups, who appear doubly disadvantaged by their illness and their socioeconomic and/or ethnic background. Three particular challenges were suggested to account for these disparities: stigma, lack of knowledge (within the general public, amongst healthcare workers and policy makers) and lack of power, particularly in improving current deficiencies. These challenges appear to be significant factors in preventing increased research funding and healthcare provision for ME generally. We call on government and funding bodies to provide strategic funding to correct years of systemic under‐resourcing. Widespread educational initiatives should alert healthcare workers and the public to the possible presence of ME in people from under‐served groups. Research is now urgently needed to understand the barriers to diagnosis and care for people with this illness, particularly for those from under‐served groups.

**Patient or Public Contribution:**

The first author, a person with ME, was a patient representative on the government‐initiated Research Working Group. As a result, she organised a series of online workshops on ME clinical research, attended by researchers, clinicians, charity representatives and people with ME. She directed the workshops and people with ME actively participated in the discussions. The last workshop examined ME research and under‐served groups. The workshop was chaired by the third author and attended by the second author. The first author conceived the article and wrote it in consultation with the second and third authors.

## 1 Introduction

Myalgic encephalomyelitis (ME) (also known as ME/CFS, CFS/ME or chronic fatigue syndrome) is a complex multi‐system chronic condition of unknown aetiology with often a poor likelihood of recovery. Clinical definitions encompass debilitating fatigue of new onset which is not relieved by rest, post‐exertional symptom exacerbation, cognitive impairment, and unrefreshing sleep. Loss of functional capacity varies with severity, from mildly affected people needing some reduction in working hours, to very severely affected being bedbound and totally dependent on carers. The UK prevalence is estimated between 250,000 and 400,000 people [1]. Amongst the nearly two million people with Long COVID in the UK, about half now fit the clinical definition for ME with biomedical and physiological similarities also being revealed, although it is still unclear if these are the same illnesses.

In many countries both clinical care and research funding for ME are grossly underfunded and under‐resourced, particularly compared to disease burden. The economic cost to the UK in 2014/15 was estimated at £3.3 billion per year. Despite this, in many parts of the UK, there are no specialist NHS ME clinics, and other hospital specialists often have little knowledge of ME. The complex needs of people with very severe ME are particularly poorly met within the NHS and care system. Many are left without support from primary or specialist care due to their extreme fatigue [2].

## 2 Involvement of People With ME in Research and Patient‐Driven Initiatives

Involvement of people living with ME in research requires major adaptations to the research process due to the severity of symptoms, which are more disabling than in many other common chronic illnesses [2]. Particular challenges are peoples' very limited energy, cognitive impairment and sensitivity to surroundings and the fluctuating nature of the condition. Enabling those most severely affected to participate is especially challenging as they may be unable to leave their beds, tolerate visitors or undertake any activity including mental stimulation except for very short periods, and this has time and cost implications.

Recently funded research studies have prioritised involvement of people with ME. For the DecodeME study, the world's largest genetic study of ME, people with lived experience and ME charity personnel were central to the grant application, design and conduct of the study. Lessons learnt from this involvement included the slow initial set‐up but consequent improvements throughout the study, and the limited ability of people with very severe ME to participate even in studies designed with them in mind.

A major participatory project, the ME/CFS James Lind Alliance Priority Setting Partnership (PSP), enabled people with ME, their carers and clinicians to prioritise research questions for ME in 2021‐22. The steering group was mainly composed of people with ME or their carers. The online questionnaires and subsequent voting for the top 10 research priorities attracted more participants than any previous PSP. Despite UK Research and Innovation highlighting these top 10+ research priorities, no applications around these questions have yet received funding from them.

A cross‐government delivery plan for ME was initiated in 2022, aiming to improve the lives of people with ME. Three working groups were set up with people with ME or carers as co‐chairs. The interim delivery plan consultation received large numbers of responses. However, no funding was provided by the last government to drive the delivery plan forward or to act on its recommendations, which remain unpublished.

The Research Working Group (RWG) was set up as part of the process, to improve the funding, amount and quality of UK ME research. The first author, a person with ME and RWG member, organised a series of four workshops focussing on clinical research in ME, including clinical trial design and drug repurposing for ME. All were well attended by researchers, clinicians, funders and people with lived experience of ME. All workshops are now available through the Action for ME YouTube account (as this charity stepped up to assist) [3].

## 3 Workshop on ME Research and Under‐Served Groups

The final workshop organised on ME research and under‐served groups was, we believe, the first UK meeting on this subject. Five speakers discussed a range of challenges focussed on ME research and under‐served groups.

**Figure 1:**
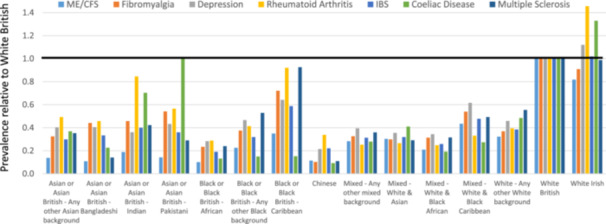
Relative prevalence of ME and other diseases stratified by ethnic group. Prevalence of ME (ICD‐10:G93.3), Fibromyalgia (ICD‐10: M79.7), Rheumatoid arthritis (M05.3, M05.8, M05.9, M06.0, M06.8, M06.9 or M08.0), clinical depression (F32 or F33 codes), Irritable Bowel Syndrome (K58.0 or K58.9), Coeliac Disease (K90.0), or Multiple Sclerosis (G35) for self‐reported ethnicities relative to ‘white British’. Codes were downloaded from NHS DigiTrials on 8th October 2023 [1]. Figure 1 is reproduced from Samms, G. L., & Ponting, C. P. (2024). Unequal access to diagnosis of myalgic encephalomyelitis in England. https://Www.Medrxiv.Org. https://doi.org/10.1101/2024.01.31.24302070. It is available under a CC‐YB license.

A major challenge appears to be significant under‐recording of ME in under‐served groups. Using Hospital Episode Statistics data, Samms and Ponting analysed the G93.3 (ICD‐10) code, ‘postviral fatigue syndrome’, (there is currently no code for ME itself) (Figure 1) [1]. Diagnostic rates varied widely by age, sex and ethnicity, with a 50‐fold difference between white females 41+ years and other‐than‐white males 0–40 years. The diagnostic rates for other‐than‐white males and females were both one‐fifth of the corresponding white group at all ages. This bias exceeds what is seen in other common diseases and was particularly marked for people of African, Chinese or Asian ethnicity. GPs practicing in the most deprived areas were least likely to record the diagnosis of ME.

It follows that requiring a confirmed ME diagnosis as an eligibility criterion for research studies will immediately limit participation by people from minoritised or socially disadvantaged communities. In the DecodeME study, only 3.3% of participants were not self‐reporting their ethnicity as White, compared to 18.3% in England and Wales who identified as ‘nonwhite’ in the 2021 census.

People with very severe ME rarely participate in research due to profound fatigue and cognitive impairment. Any involvement carries risks of harming individuals by precipitating worsening of already precarious existences. One simple questionnaire for people with very severe ME was described, successfully using appropriate adaptations and flexibility, for example, having no deadline to complete the questionnaire (in practice, from 3 days to 1 year). The resulting publication, case histories of five people requiring tube feeding, showed serious defects in care, such as allowing people to remain severely malnourished, inappropriately diagnosing psychological conditions and inordinately long delays before initiating enteral feeding [2].

Other speakers highlighted barriers to effective healthcare for people from under‐served groups, such as racism‐based trauma from healthcare workers in young people with sickle cell, and limited awareness of Long COVID and previous negative experiences of healthcare delaying help‐seeking in people with probable Long COVID. Similar factors may be responsible for low diagnostic and referral rates in people with ME from under‐served groups although there is very little published research around this. In a series of papers from Manchester in 2013 to 2014, barriers were found at every stage to the diagnosis and management of CFS/ME in people from Black and minority ethnic groups [4].

## 4 Key Challenges Identified

Several themes recurred throughout the workshop; stigma, lack of knowledge and the lack of power to change existing narratives and so influence health and research agendas.

### 4.1 Stigma

Stigma is a contributory factor in poor health care experiences, inducing trauma for people with ME [5]. This is compounded by the lack of a diagnostic test or any licensed treatments. Stigma leads to self‐doubt and contributes towards lack of help‐seeking. Stigma towards people with ME, particularly those severely affected and/or hospitalised, can prevent adequate assessments and care [2]. People from under‐served groups seeking healthcare experience stigma for a variety of reasons, including racism and stigma arising from social disadvantages, and these probably contribute to the very low diagnostic rates for ME in these individuals (Figure 1) [1].

### 4.2 Lack of Knowledge

Lack of knowledge about ME is widespread amongst health care professionals, amongst many communities and policy makers. It contributes to delayed diagnosis (which is typically several years from onset of symptoms [4]) or lack of diagnosis, as seen in underserved groups [1]. Previous aetiological hypotheses of deconditioning or psychosomatic causation and denial of the person's illness experience led to years of epistemic injustice and still appear to underlie many poor healthcare decisions [5]. People with very severe ME needing tube feeding were often inadequately treated due to profound ignorance by health care professionals [2].

The differing diagnostic rates across England for ‘post viral fatigue syndrome’ suggest that many GPs are unaware of the accepted diagnostic criteria for ME [1]. This was particularly true for GPs serving areas of multiple deprivation or minoritised communities. Several published surveys of GPs and other healthcare workers have confirmed low levels of knowledge and widespread misconceptions about ME.

### 4.3 Lack of Power

Despite the high cost to government and to society, post‐viral illnesses and fatigue appear a low priority for those in power. Previous UK governments' beliefs that individuals were responsible for their own success or failure side‐lined those least able to advocate for themselves, including those with energy‐limiting conditions, particularly those from under‐served groups. During the recent COVID‐19 pandemic, government planning ignored the possibility of long‐term post‐viral fatigue emerging despite previous pandemics resulting in ME‐like syndromes.

Communication with healthcare professionals is challenging for people with ME. Fatigue and cognitive impairment limit the ability to self‐advocate, while the illness itself is characterised by multiple fluctuating symptoms which are difficult to describe. Additional barriers for people from under‐served groups may be systemic arising from socioeconomic status or institutional racism, but could also be individual, arising from language and culture or specific communication difficulties such as deafness or learning disabilities [4]. Those who are severely ill can find any communication extremely taxing [2]. In all these situations, the person with ME experiences powerlessness, which affects their ability to access effective healthcare.

ME continues to receive low levels of research funding from public funders in many countries. The UK government has recently reiterated that no additional funding is available for ME research. Connected with the proposed delivery plan, UK Research and Innovation acknowledged the relatively low amount of biomedical research compared to disease burden, the lack of trust between different stakeholders and low current research capacity. The interim delivery plan stated the Medical Research Council and National Institute for Health and Care Research have funded £8.05 million in grants over the past 10 years. This amounts to £2.88/person with ME out of £74/person/year of public sector funding of health research. Government and funders do make exceptions to enable funding of particular disease areas, so their power can override constrained finances whenever the political will exists.

Coproduction and power‐sharing, often necessitating changes in study design or timing, also fit poorly with funders' requirements for fully costed and timed studies. Coproduction is underpinned by ideas of sharing power and knowledge and, as in DecodeME, improves outcomes when effective. In contrast, there have been repeated delays to the UK government delivery plan for ME, where power resides within government.

### 4.4 The Way Forward

Improving clinical care for ME starts with education of all healthcare professionals during training and continuing education, professional bodies including the Royal Colleges, and policy makers. The recent creation of educational modules for healthcare practitioners coordinated by NHS England is welcome but needs to be widely disseminated and may take time to become embedded.

Public awareness campaigns through a range of channels such as GP surgeries and the media would reduce stigma and increase awareness of the disease, enabling people to consult about troubling symptoms which may be ME. The campaign should also focus on the universal nature of ME and particularly its presence in people from under‐served groups.

All patients with ME should have access to local multi‐disciplinary specialist clinics, which are lacking in many countries. In the UK this needs government initiatives and funding to enable widespread implementation of the NICE 2021 guidance (NG206). Those severely affected need local and regional provision along with updated national guidelines in specific areas such as nutrition, backed by expert consensus and research where feasible.

The low historic funding of ME research has led to a paucity of research on ME clinical care and provision in the UK. More research into improving the quality of care is urgently needed. In addition, although there is currently no cure for ME, repurposed drugs and other therapies showing promise and used extensively in other countries need trialling, such as low‐dose naltrexone, treatments for mast cell activation syndrome and orthostatic hypotension. As research will take time, interim measures could include listings of these drugs as unlicensed indications in the British National Formulary and specialist ME clinics using therapies off‐license, with pre‐and post‐monitoring to understand the characteristics of responders.

Decisions made by funding body committees in various countries, including the UK, suggest that stigma and misinformation can still influence reviews of grant applications. Strategies directly addressing this issue need country‐specific solutions, such as a specific panel to review ME grant applications, or educational initiatives for funding committees.

Much research is needed to understand the impact of ME in under‐served groups and reasons for the varying diagnostic rates, including potential barriers in different groups [1, 4]. Pilot studies could trial methods to boost diagnosis and care relevant to different situations, designed and conducted in partnership with local communities.

Improving involvement in research of people with ME from under‐served groups will be problematic until ME becomes more widely diagnosed. Initiatives in other disease areas may inform but need tailoring to the disabling symptoms of ME. Specific measures include situating recruitment within acceptable local sites and recruiting people with symptoms rather than a confirmed diagnosis. In the latter case, only those then receiving a diagnosis of ME by a clinician would be enroled. This strategy may also enable more people from under‐served groups to be diagnosed with a range of fatiguing illnesses, including ME, and to receive relevant healthcare advice.

Initiatives in other disease areas have included both funders and journals requiring more information to demonstrate that equality, diversity and inclusion have been adequately considered by researchers and reported upon. Published research on ME has omitted the ethnicity of participants even when available, for example, when using UK Biobank data, and little published research has specifically included people from minority communities. Requiring information on the ethnicity and social status of participants would raise awareness of these issues amongst researchers.

The economic case to fund ME research is indisputable. Providing adequate funding for ME research could be cost‐effective within the terms of this UK parliament. DecodeME may reveal new avenues for research. Strategic funding would support rapid development of diagnostic tests, treatment pathways and understanding of the aetiology of ME within a short timescale. It would also open the way for improved diagnosis and healthcare for those with ME from under‐served groups.

## 5 Conclusion

Ongoing stigma, lack of knowledge, and particularly of power are preventing adequate provision of health care and research for people with ME. The consequences of this are particularly stark for those from under‐served groups who are less likely to be diagnosed with ME and almost never involved in research. Government initiatives are needed, including the long‐awaited delivery plan which aims to improve both care and research. All initiatives need to be backed by adequate finance. Strategic funding is called for, directed at ME research, widespread educational initiatives and adequate funding of health and social care. The perception of ME needs to change, from a disease where only those able to advocate for themselves are diagnosed, to acceptance that ME can be highly disabling and likely to affect under‐served groups particularly hard.

We did not intend this paper to be just a long list of complaints but aim to show how people with ME could join the decision‐making process.

## Author Contributions

**Monica Jane Bolton:** conceptualisation, writing – original draft, writing – review and editing. **Carolyn A. Chew–Graham:** writing – original draft, writing – review and editing. **Harm Marwijk:** writing – original draft, writing – review and editing.

## Ethics Statement

The authors have nothing to report.

## Conflicts of Interest

Carolyn Chew‐Graham is Editor in Chief of Health Expectations. The views expressed are those of the author(s) and not necessarily those of the NHS, the NIHR or the Department of Health and Social Care.

## Data Availability

No original data is presented in this paper. The video recording of the online workshop on ME research and under‐served groups can be viewed on the Action for ME YouTube channel, see link in references.
